# PREPARE: Pre-surgery physiotherapy for patients with degenerative lumbar spine disorder: a randomized controlled trial protocol

**DOI:** 10.1186/s12891-016-1126-4

**Published:** 2016-07-11

**Authors:** Yvonne Lindbäck, Hans Tropp, Paul Enthoven, Allan Abbott, Birgitta Öberg

**Affiliations:** Department of Medical and Health Sciences, Division of Physiotherapy, Faculty of Health Sciences, Linköping University, SE-58183 Linköping, Sweden; Department of Clinical and Experimental Medicine, Linköping University, Linköping, Sweden; Faculty of Health Science and Medicine, Bond University, Gold Coast, QLD 4229 Australia

**Keywords:** Low back pain, Physiotherapy, Stratification, Surgery, Function

## Abstract

**Background:**

Current guidelines for the management of patients with specific low back pain pathology suggest non-surgical intervention as first-line treatment, but there is insufficient evidence to make recommendations of the content in the non-surgical intervention. Opinions regarding the dose of non-surgical intervention that should be trialled prior to decision making about surgery intervention vary. The aim of the present study is to investigate if physiotherapy administrated before surgery improves function, pain and health in patients with degenerative lumbar spine disorder scheduled for surgery. The patients are followed over two years. A secondary aim is to study what factors predict short and long term outcomes.

**Methods:**

This study is a single blinded, 2-arm, randomized controlled trial with follow-up after the completion of pre-surgery intervention as well as 3, 12 and 24 months post-surgery. The study will recruit men and women, 25 to 80 years of age, scheduled for surgery due to; disc herniation, spinal stenosis, spondylolisthesis or degenerative disc disease. A total of 202 patients will be randomly allocated to a pre-surgery physiotherapy intervention or a waiting list group for 9 weeks. The waiting-list group will receive standardized information about surgery, post-surgical rehabilitation and advice to stay active. The pre-surgery physiotherapy group will receive physiotherapy 2 times per week, consisting of a stratified classification treatment, based on assessment findings. One of the following treatments will be selected; a) Specific exercises and mobilization, b) Motor control exercises or c) Traction. The pre-surgery physiotherapy group will also be prescribed a tailor-made general supervised exercise program. The physiotherapist will use a behavioral approach aimed at reducing patient fear avoidance and increasing activity levels. They will also receive standardized information about surgery, post-surgical rehabilitation and advice to stay active. Primary outcome measure is Oswestry Disability Index. Secondary outcome measures are the visual analogue scale for back and leg pain, pain drawing, health related quality of life, Hospital anxiety and depression scale, Fear avoidance beliefs questionnaire, Self-efficacy scale and Work Ability Index.

**Discussion:**

The study findings will help improve the treatment of patients with degenerative lumbar spine disorder scheduled for surgery.

**Trial registration:**

ClinicalTrials.gov reference: NCT02454400 (Trial registration date: August 31st 2015) and has been registered on ClinicalTrials.gov, identifier: NCT02454400.

## Background

Low back pain (LBP) have a lifetime prevalence to up to 84 % and is a major cause of disability with substantial socioeconomic impact globally [1]. The highest prevalence is in age-group 45–64 and many experience functional impairments and activity limitations. Up to 85 % of LBP is considered to have non-specific etiology [2] when the mechanism for LBP cannot be clearly identified. LBP can be the product of nociception from lumbar spine structures [3] or even the result of centrally mediated pain in the absence of nociception [4]. Different LBP related pathologies can coexist but approximately 5 % of those receive a primary diagnosis of disc herniation [5], 3–4 % spinal stenosis [6] and lower number with spondylolisthesis and degenerative disc disease (DDD). In Swedish Spine register of spinal surgery patients these diagnoses are concluded as degenerative lumbar spine disorders [7].

Guidelines published internationally focusing on the management of LBP in primary care recommend screening for serious pathology, neurological symptoms, the consideration of psychosocial risk factors if there is no improvement and to avoid routine imaging for non-specific LBP [8]. The prevalence of serious pathology in the form of malignancy, spinal fracture, infection, or cauda equine syndrome requiring referral to secondary or tertiary medical care occurs in only <1–4 % of primary health care LBP cases [9–11]. Guidelines recommend a multicomponent strategy for patients with longstanding LBP including supervised exercises and behavioral approach based on the presence of yellow flags [8].

Consistencies in the patient’s history, physical assessment, clinical tests and medical imaging may strengthen suspicion of specific pathologies such as disc-herniation, spinal stenosis, spondylolisthesis and DDD causing LBP [12–15]. In the absence of serious pathology, disc herniation usually has a good prognosis and non-surgical intervention is recommended 8–12 weeks before decision-making about surgery [16–18]. Leg pain intensity is a significant prognostic factor for subsequent surgery after non-surgical intervention [14]. A recent overview of the literature suggests that surgery leads to short-term benefits for leg pain and to a lesser extent for LBP when compared with non-surgical treatment. Despite this, no short-term and long term effects have been observed for functional outcome measures [5]. It is also uncertain if surgical intervention has positive or negative effects on the underlying disc disease in a longer perspective [5]. In disc herniation, similar results have been observed for non-surgical and surgical intervention at one [5, 18] and two years follow-up [5], which supports the view that the non-surgical intervention should be thoroughly tested before decision making about surgery.

In mild to moderately symptomatic degenerative lumbar stenosis, the prognosis can be favorable in up to half of patients and non-surgical intervention is recommended before decision-making about surgery [19]. However, a recent Cochrane review suggests that, high quality studies are needed to inform future evidence based guidelines about the content of the non-surgical intervention [20]. The prevalence of spinal stenosis is increasing and therefore high quality research its management is of importance [20]. In the older population spinal stenosis is the most common reason for spinal surgery. Surgical intervention is recommended in moderate to severe spinal stenosis [19], but up to 35 % of the patients remain doubtful or dissatisfied with the result of surgery [7].

Current guidelines for the management of degenerative lumbar spondylolisthesis suggest that when there is a predominance of stenotic radicular symptoms, treatment should be similar to treatment for symptomatic degenerative lumbar spinal stenosis [21]. Therefore, surgical intervention is recommended when symptomatic spinal stenosis associated with low grade degenerative lumbar spondylolisthesis is recalcitrant to trial of non-surgical intervention [20]. There is currently a limited evidence base comparing the two interventions for spondylolisthesis suggesting surgical intervention to be more successful than non-surgical intervention for pain and functional outcomes [22].

In patients with DDD, both non-surgical and surgical interventions have limited evidence [18]. In a review of systematic reviews and RCTs, Jacobs et al [18] reported that for DDD, surgery is no more effective than high-intensity non-surgical interventions for improvements in pain scores or function. Similarly, a recent systematic review and meta-analysis suggests that there is strong evidence that lumbar fusion surgery is not more effective than non-surgical interventions in reducing disability because of chronic LBP [23].

Previous literature on the treatment of LBP has often been based on generic treatments for patients with heterogeneous body functional impairments and activity limitations. This may explain the small effect sizes reported in clinical trials [24]. Recently attention has shifted to the homogenous classification of patients based on functional impairments and activity limitations with the aim of improving the effectiveness of individualized treatments [25]. This has led to a focus on stratified care for LBP in the area of physiotherapy [25]. One of these stratification care models is the Treatment Based Classification (TBC), using criteria from the subjective and physical assessment to classify patients to different treatment classifications [26]. TBC has shown better treatment outcome than treatment according to guidelines in acute LBP [27], but this effect has not been found in patients with long-lasting non-specific LBP [28].

High quality evidence-based guidelines for the non-surgical intervention of these degenerative lumbar spine disorders are lacking. The optimal duration of non-surgical intervention before surgical intervention is considered unclear. For those patients selected for surgical intervention, there is currently a small body of literature suggesting that pre-surgery physiotherapy improves the outcome of spinal surgery [29, 30]. Nielsen et al showed that supervised home exercise to improve trunk muscle strength and cardiovascular conditioning gave better post-operatively functionality, faster recovery and shorter hospital stay. The patients reported higher level of satisfaction in the intervention group [29]. Louw et al tested the effect of pre-surgery education, which showed lower health care consumption and a more favorable surgical experience [30]. No study has tested a more comprehensive exercise program pre-surgery. In hip pathology, physiotherapy prior to joint replacement surgery has been shown to reduce pain and improve physical function in preparation for surgery [31]. The extent to which patients have access to non-surgical intervention prior to decision-making about surgery is also lacking in the literature. Only 10 % of the clinical studies about the effect of surgery have information about non-surgical intervention prior to surgery for spinal stenosis [32]. We hypothesize that supervised exercises that have been proposed for non-specific LBP combined with a TBC could be effective also for patients awaiting elective surgery. The prognosis of LBP is multifactorial and therefore it is of interest to understand other factors apart from pre-habilitation that can influence short- and long term outcome.

### Objectives

The aim is to investigate if physiotherapy administrated before surgery improves function, pain and health in patients with degenerative lumbar spine disorder scheduled for surgery. The patients are followed over two years. A secondary aim is to study what factors predict short and long term outcomes.

### Trail design

This study protocol describes a single blinded, 2-arm, randomized controlled trial with 2 year follow up and the 1 year follow up as primary end point. The protocol conforms to the SPIRIT 2013 recommendations.

## Methods

### Study setting

A total of 202 consecutively selected patients will be included in the study. All patients are referred to the Spine Clinic at the University Hospital in Linkoping, Sweden. Individuals that fulfil the inclusion criteria will be asked to participate. After signed informed consent has been obtained, baseline measurement will be collected and randomisation will take place.

### Eligibility criteria

Inclusion criteria; Males and females aged 25–80 years that are scheduled for surgery with degenerative lumbar spine disorder; presence of low back and/or leg pain due to disc herniation, spinal stenosis, spondylolisthesis (Grade 4), degenerative disc disease (DDD); diagnosis confirmed by magnetic resonance imaging; pain level high enough to indicate surgical intervention; fluent in Swedish.

Exclusion criteria: Patients that are in need of acute surgery; have severe spinal pathology (such as osteoporosis, or fusion > 4 levels (5 vertebras) or other severe diagnoses; patients in need of re-surgery on the same level.

### Interventions

#### Pre-surgery waiting list group

Patients will receive standardized information about surgery from an orthopedic surgeon, post-surgery rehabilitation and advice to stay active.

#### Pre-surgery physiotherapy group

Patients will receive pre-surgery physiotherapy intervention twice a week for 9 weeks. The program is multicomponent and will include:

1. Active physiotherapy according to a TBC; a) Specific exercises and mobilization, or b) Motor control exercises or c) Traction.
2. Tailor-made general supervised exercise program.
3. Behavioral approach to reduce fear avoidance and increase activity level.

Patients will also receive standardized information about surgery from an orthopedic surgeon, post-surgery rehabilitation and advice to stay active.

The physiotherapy intervention will be performed at one of eleven different physiotherapy clinics in Östergötlands county, close to the patient’s home. The physiotherapists delivering the intervention program will be trained by two specialist physiotherapists with two initial meetings and three follow ups during the study. For each patient the physiotherapist will follow a checklist with treatment and progression planned for each treatment-session, but modified to suit the patient. The content in the exercise-program will include 10 min intervals of cardiovascular-exercise in the beginning, in the middle and at the end of each session. Further it will include 5–6 exercises based on the patients’ function, posture and PSFS (patient specific functional scale) with the dosage of 15 repetitions in three sets. Variation of exercise equipment and consideration of the need of different starting positions is recommended and if possible the prescription of some exercises in upright standing. At least one exercise will be altered every third visit, both with aim of progressing the training as well as providing a distractive pain coping strategy. The Borg scale [33] will be used for rating the perceived exertion of each exercise session. The prescribed level of exertion for each exercise session will be 12–13 on Borg Scale. If the patient score lower than 12–13 on the Borg scale, the patient will be informed to increase the intensity of the exercises. The patients will also receive written and illustrated descriptions of exercises and their dosage in the exercise-program. At home, the patients will complete a logbook of self-mediated home exercise and general physical activities. A treatment demarcation table will be provided outlining the intervention content for each group (Table 1). Checklists and logbooks will provide data regarding adherence. The physiotherapist will urge the patients to continue the treatment program and the reason for terminating will be noted. A minimum intervention quota of 50 % will be required to be fulfilled for the intervention to be considered as compliant. Spinal surgery and post-surgery rehabilitation will be performed according to clinical routine [5].

**Table 1:** Components of the two intervention protocol used in the trial

Treatment components	Waiting list group	Pre-surgery physiotherapy group
1a. Specific movement and mobilisation - Mechanical loading strategies; repeated movements according to directional preference, mobilisation, and integration in a functional restoration program.	**X**	**TBC**
1b. Motor control exercise - Exercise focused on trunk and pelvic floor muscle activation and integration in a functional restoration program	**X**	**TBC**
1c. Traction - Manual Traction producing symptom reduction	X	**TBC**
2. Tailor-made general supervised exercise program - Individualised prescription (type of exercise and load) of aerobic, resistance, flexibility exercises with information documents and checklist for treatment, progression and dosage	X	**✓**
3. Behavioral approach: - Goal setting (establishment and regular reviews) - Strategies to minimize barriers to goal attainment, for example education regarding; ergonomics, postural alignment, patho-anatomical/physiological explanation - Self-mediated home exercise and physical activities (logbook)	X	**✓**
Advice regarding general physical activity recommendations	**✓**	**✓**
Education regarding surgery	**✓**	**✓**
Education regarding post-surgical rehabilitation	**✓**	**✓**
Pre-surgical re-evaluation	**✓**	**✓**

Spinal surgery and post-surgery rehabilitation in both groups is performed according to existing clinical routine [5]

✓ component mandatory, ***X*** component not allowed, ***TBC*** treatment based classification component with modifications

### Outcomes

All questionnaire based outcome measures will be performed at baseline, before surgery (after pre-surgery physiotherapy or waiting-list), as well as three months, one and two years after surgery. Two written reminders and if needed one telephone call will be used to enhance response rate. Subjective, physical assessment measures and TBC will be collected at baseline and after completion of the 9 weeks’ intervention.

#### Primary outcome measure

The primary outcome measure will be patient self-reported function and activity limitation measured by Oswestry Disability Index (ODI) [34]. ODI contains ten items investigating pain related function impairment and activity limitations with six answer alternatives for each item. The sum score is between 0-100 %, with higher values representing higher functional disability score [34].

#### Secondary outcome measures

Pain will be evaluated with VAS [35], pain drawing and pain duration [36]. Health related quality of life will be evaluated with SF-36 [37] and EQ-5D [38]. Anxiety and depression will be evaluated with the Hospital Anxiety and Depression Scale (HADS) [39] and self-efficacy with Self-efficacy scale (SES) [40]. Fear avoidance will be measured by Fear avoidance belief questionnaire (FABQ) [41]. Work will be assessed by general information of work and Work Ability Index (WAI) [42]. Patient reported treatment effects will be measured by Patient reported global treatment effects and patient enablement instrument (PEI) [43]. Expectations, sick-leave, lifestyle behavior, previous healthcare consumption and adverse events will also be measured. Patients randomized to pre-surgical physiotherapy will be evaluated with the Patient Specific Functional Scale (PSFS) [44] three times during the intervention. Subjective, physical assessment measures and TBC will be used as secondary outcome measures.

### Participant timeline

**Fig. 1:**
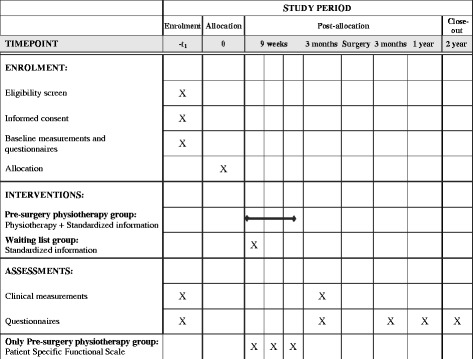
Time schedule of enrolment, interventions and assessment

The study design is outlined in Fig. 1.

### Sample size

Based on a minimally clinical important change of 10 % on the ODI [45], a standard deviation for the ODI =20, a significance level of *p* = 0.05, a power of 80 %, an estimated 64 patients are required in each of the intervention groups, at the primary endpoint. The current sample size of 202 patients allows for a 35 % loss to follow-up while maintaining >80 % statistical power.

### Allocation

Block randomization will be used with smaller block size at the end of the study. Initially two blocks of 60 will be created and then subsequent blocks of 20, 8 and 4. For each randomization block, sealed opaque envelopes will be prepared with a 1:1 ratio of allocation to the pre-surgery waiting list group as well as the pre-surgery physiotherapy group. After baseline measurement, an independent physiotherapist working at the Spine Clinic will open a sealed opaque randomization envelope and inform the patient about group allocation. The same physiotherapist will contact the physiotherapy center where the patient will receive treatment.

### Blinding

The physiotherapist performing the clinical measures and assigning a treatment based classification will be blinded to the randomization, while patients and the physiotherapists treating the patients cannot be blinded.

### Data collection methods

#### Clinical tests and TBC procedure

Baseline subjective and physical assessment measures will be assessed by a physiotherapist. The same assessment will be conducted at follow up after the intervention. The subjective assessment will include information on symptom duration, prior LBP, distribution of symptoms, symptom aggravating and easing positions (sitting, standing or walking), time tolerated in positions (sit, stand and walk) as well as signs of centrally disturbed pain modulation. Physical assessment will include test for aberrant movements, neurological tests (myotomes, dermatomes and reflexes) for spinal segmental levels L4-S1, straight leg raising, range of motion for internal rotation of hips, posterior-anterior-test lumbar segmental provocation test (PA-test), Sacroiliac joint provocation tests (SI-tests), test for symptom centralization, prone instability test, active straight leg raise (A-SLR), Trendelenburg test, isometric bilateral quadriceps strength and 10 m walking-test [46, 47].

**Fig. 2:**
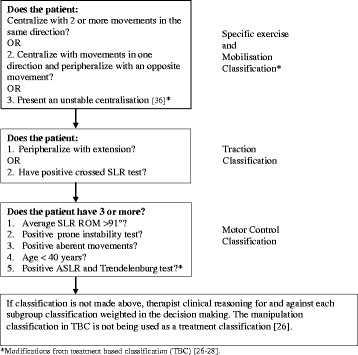
Treatment based classification (TBC) [26–28] used in the trial

The following TBC will be utilized; a) Specific exercises and mobilization, b) Motor control exercises or c) Traction. The TBC criteria for patient’s subjective and physical examination findings which has been outlined in previous publications [26–28] will be utilized except for the “Specific exercises” and “Manipulation” classifications in the original TBC being collapsed to one classification named “Specific exercises and mobilization”. This means that manipulation will not be used as a treatment classification. The reason for this is that the patients included in the study have long-lasting pain that does not fulfil the TBC criteria of symptom duration < 16 days for the manipulation classification [27]. Furthermore, literature suggests that 25 % of the patients fulfill criteria for more than one classification and for 68 % of these cases it is the combination of criteria for “Specific exercises” and “Manipulation” [26]. In the “Specific exercise and mobilization” classification, a test for centralization with lumbar spine flexion combined with rotation in side lying position will be added, to assess more directions than only sagittal plane movement. This position is commonly used in clinical practice for assessment and treatment of patients with sciatica. If repeated exercises only give an unstable centralisation, where the pain is reduced or abolished during the repeated movement testing or positioning but after resuming a weight bearing position for one minute, the pain intensity level returned to the pre-testing intensity, patients will be classified to this classification [48]. This added criteria is useful in patients who have significant activity limitation due to pain, are too deconditioned to perform repetitions of tests or are patients who activity limiting symptoms are not painful such as in some spinal stenosis cases. In the PA-test, segmental pain will be documented, but not hypo- or hypermobility, due to lack of reliability in segmental motion restriction [49]. The tests “Active straight leg raising” (ASLR) and Trendelenburg will be added as criteria for the “Motor control exercises” classification [28]. The TBC is outlined in Fig. 2.

### Data management

Data will be stored at the research department at Linköping University, not allowing for identification for individuals.

### Statistical methods

Data collected at the longitudinal time points will be analysed according to the ‘intention to treat’ (ITT) principle. Data analysis according to ITT requires that data for every patient, regardless of level of intervention compliance, data missing at follow-up or due to drop-out, will be included in the group to which they are randomised [50]. To perform an ITT analysis, missing data will be replaced through multiple imputation methods. In an attempt to determine the sensitivity of the ITT data, it will be compared to per-protocol data from patients who have complied with the original study protocol. Alternative analyses will also be performed to take treatment compliance into consideration. Analysis of variance ANOVA will be used to investigate within and between group differences in longitudinal outcome measures. Within and between group Cohens *d* effect sizes will also be calculated. Multivariate regression will be used to analyse potential predictors of long term outcomes.

## Discussion

In degenerative lumbar spine disorders the outcome after surgery is questioned since about 20–35 % are doubtful or dissatisfied with the results at one year follow up [7]. Few studies have used physiotherapy as a preparation before surgery, one with information and one with home exercise program that improved patient reported outcome and healthcare consumption [29, 30]. The question still remains if more comprehensive physiotherapy intervention will change the short- and long term outcome. In this study we target several dimensions in the biopsychosocial model. It is known that there is a risk that patients with persistent pain develop fear avoidance and maladaptive pain behavior, deterioration in physical performance and inactivity. In contrast to previous studies the strategy is based on the TBC and tailor-made exercise program. This does not allow to study single interventions. The strength is to introduce a standardized assessment for this patient group that can be part of a clinical reasoning process.

The design includes a broad assessment that will make it possible to look for predictive factors in this heterogeneous population supporting a better risk assessment of good or poor outcome.

A limitation of the study is that the treatments cannot be blinded to patient and treatment provider. The generalizability of the study’s results may be affected by the process of selecting patients for surgery which might be different in different countries and health care systems. Another limitation might be that all patients will be informed that they are scheduled for surgery, which could influence their expectations and attitudes towards pre-surgery-physiotherapy and bias self-reported outcome.

### Trial status

The trial is ongoing and the final two-year follow-up will be finished November 2018.

### Summary

This study uses a randomized controlled design to investigate if pre-surgery physiotherapy improves function, pain and health in patients with degenerative lumbar spine disorder before and after surgery. The study will also investigate what factors predict short and long term outcomes. The novel findings will contribute to evidence-based recommendations as to the effect of stratified pre-surgery physiotherapy in patients with degenerative lumbar spine disorder scheduled for surgery. Furthermore, findings will provide direction for future research.
